# Effects of short-term fasting on *in vivo* rumen microbiota and *in vitro* rumen fermentation characteristics

**DOI:** 10.5713/ajas.18.0489

**Published:** 2018-09-13

**Authors:** Jong Nam Kim, Jaeyong Song, Eun Joong Kim, Jongsoo Chang, Chang-Hyun Kim, Seongwon Seo, Moon Baek Chang, Gui-Seck Bae

**Affiliations:** 1 Deparment of Animal Biosystem Sciences, Chungnam National University, Daejeon 34134, Korea; 2 Department of Food Science & Nutrition, Dongseo University, Busan 47011, Korea; 3 Department of Animal Science, Kyungpook National University, Sangju 37224, Korea; 4 Department of Agricultural Science, Korea National Open University, Seoul 03087, Korea; 5 Department of Animal Life and Environmental science, Hankyung National University, Anseong 17579, Korea; 6 Department of Animal Science and Technology, Chung-Ang University, Anseong 17546, Korea

**Keywords:** Holstein Steers, *In vitro*, Fasting, Rumen Microbiota, Denaturing Gradient Gel Electrophoresis

## Abstract

**Objective:**

Fasting may lead to changes in the microbiota and activity in the rumen. In the present study, the effects of fasting on rumen microbiota and the impact of fasting on *in vitro* rumen fermentation were evaluated using molecular culture-independent methods.

**Methods:**

Three ruminally cannulated Holstein steers were fed rice straw and concentrates. The ruminal fluids were obtained from the same steers 2 h after the morning feeding (control) and 24 h after fasting (fasting). The ruminal fluid was filtrated through four layers of muslin, collected for a culture-independent microbial analysis, and used to determine the *in vitro* rumen fermentation characteristics. Total DNA was extracted from both control and fasting ruminal fluids. The rumen microbiota was assessed using denaturing gradient gel electrophoresis (DGGE) and quantitative polymerase chain reaction. Microbial activity was evaluated in control and fasting steers at various intervals using *in vitro* batch culture with rice straw and concentrate at a ratio of 60:40.

**Results:**

Fasting for 24 h slightly affected the microbiota structure in the rumen as determined by DGGE. Additionally, several microorganisms, including *Anaerovibrio lipolytica*, *Eubacterium ruminantium*, *Prevotella albensis*, *Prevotella ruminicola*, and *Ruminobacter amylophilus*, decreased in number after fasting. In addition, using the ruminal fluid as the inoculum after 24 h of fasting, the fermentation characteristics differed from those obtained using non-fasted ruminal fluid. Compared with the control, the fasting showed higher total gas production, ammonia, and microbial protein production (p<0.05). No significant differences, however, was observed in pH and dry matter digestibility.

**Conclusion:**

When *in vitro* techniques are used to evaluate feed, the use of the ruminal fluid from fasted animals should be used with caution.

## INTRODUCTION

*In vitro* feed evaluation using small tubes and ruminal fluid has been extensively used to examine ruminant diets in the academia and industry [1]. Such techniques are based on the ruminal fluid obtained from live animals equipped with permanent rumen cannulae and require several steps, including the removal of feed particles via muslin, cheesecloth, or centrifugation. Microbial activity is assumed to reach a peak after feeding; accordingly, numerous studies have examined the rumen contents collected post feeding [2–4]. However, some studies have noted that the ruminal fluid might mask the true effect of feed or feed additives if these effects are large. Therefore, the rumen contents are collected prior to feeding (i.e., before morning feeding) [5,6]. However, nutrient perturbation, even for short intervals, influences the rumen microbiota and microbial activity in the rumen [7]. In many countries where cannulated animals are seldom available owing to their maintenance costs, it is not uncommon for scientists to obtain the rumen contents only at an abattoir where the animals are fasted without feed and water for up to a day before they are slaughtered [8]. Therefore, one might speculate that the rumen contents obtained from animals after such short-term changes in status (i.e., fasting for a day) could affect the feed evaluation process. Unfortunately, little information is known about how the microbiome responds to short-term fasting. Fasting could cause microorganisms in the rumen to encounter decreased nutrients and habitat resources as well as increased competition for food. Therefore, fasting may lead to changes in the microbiota and activity in the rumen. The aim of the present study was to evaluate the potential effects of short-term starvation on rumen microbiota using *in vivo* molecular culture-independent methods as well as the effects on *in vitro* fermentation characteristics using the ruminal fluid obtained from animals that were fasted at least for 24 h.

## MATERIALS AND METHODS

This study was approved by the Institutional Animal Care and Use Committee at the Chung-Ang University, Seoul, Korea (No. 2013-0047).

### Animals and experimental design

The representative rumen contents were obtained from three cannulated Holstein steers (793±8 kg) 2 h after morning feeding (control). Then, fasting was induced by withdrawing both feed and water for 24 h, and the rumen contents were obtained (fasting) from the same steers. The steers were offered typical commercial concentrates and rice straw at a ratio of 40:60. The rumen contents were filtrated through four layers of muslin, immediately sampled (50 mL), and snap-frozen for microbial analysis. Approximately 1 L of filtrated ruminal fluids was stored in individual Thermos bottles and transported to the laboratory for *in vitro* experiments.

### Isolation and purification of DNA

For molecular microbial analyses, DNA was isolated from the ruminal fluid samples that were collected before (control) and after fasting (fasting) from steers using a previously described method [9]. Briefly, genomic DNA was extracted by bead-beating using a Mini Bead-beater (BioSpec Products, Bartlesville, OK, USA) for 4 min at full speed in the presence of 0.7 g of zirconium beads (0.1 mm in diameter), 282 μL of Buffer A (0.2 M NaCl, 0.2 M Tris, and 0.02 M ethylenediaminetetraacetic acid [EDTA]; pH 8), 26.8 μL of Buffer PM (QIAquick 96 PCR Purification Kit, Qiagen, Valencia, CA, USA), 200 μL of 20% sodium dodecyl sulfate, and 550 μL of a phenol-chloroform-isoamyl alcohol mixture (25:24:1 by volume, pH 8). After centrifugation (16,000×*g* for 20 min at 4°C), the supernatant was thoroughly mixed with 650 μL of Buffer PB (Qiagen, USA), and the DNA sample was purified using the Qiagen PCR Purification Kit according to the manufacturer’s protocol.

### Denaturing gradient gel electrophoresis analysis

To perform denaturing gradient gel electrophoresis (DGGE) analysis, 16S rRNA gene fragments of the V3 region were amplified using the primers GC-clamp-341f (5′-TCC TAC GGG AGG CAG CAG-5′) and 518r (5′-ATT ACC GCC GCT GCT GG-3′) as described previously [10,11]. Polymerase chain reaction (PCR) was performed using a TaKaRa Bio instrument (PCR Thermal Cycler, Otsu, Japan) in a final volume of 25 μL with EmeraldAmp (GT PCR Master Mix, TaKaRa Bio, Japan), 1 μL of each primer (GC-clamp 341f and GC-clamp 354r), 2 U of *Taq* polymerase (Ex Taq, TaKaRa Bio, Japan), and 1 μL of template. After the initial denaturation at 94°C for 5 min, amplification consisted of 30 cycles of denaturation (at 94°C for 30 s), annealing (at 55°C for 30 s), extension (at 72°C for 30 s), and final extension step (at 72°C for 7 min). The PCR product was checked using 2% agarose gel electrophoresis and visualized using a Gel Doc System (Bio-Rad, Hercules, CA, USA). PCR products were concentrated and purified using the QIAquick PCR Purification Kit (Qiagen, USA). The DGGE was conducted using the D-Code System (Bio-Rad, USA) with 8% (w/v) polyacrylamide gels containing a 40% to 65% denaturant gradient, 1 mm thick, in 1×Tris acetate-EDTA buffer. Equal amounts of purified PCR products were loaded on the gel, and electrophoresis was performed at 25 V for 15 min and then 70 V for 16 h and 30 min at 60°C. The gel was stained in 250 mL of running buffer containing ethidium bromide (50 μg/mL) for 15 min. The stained gels were photographed under UV light using the Gel Doc XR documentation system (Bio-Rad, USA). The normalization and analysis of gel profiles were conducted using the XLSTAT program (Addinsoft, New York, NY, USA).

### Quantitative polymerase chain reaction

Populations of *Fibrobacter succinogenes*, *Ruminococcus albus*, *Streptococcus bovis*, *Prevotella ruminicola*, *Prevotella albensis*, *Eubacterium ruminantium*, *Anaerovibrio lipolytica*, *Ruminococcus flavefaciens*, methanogenic archaea, general protozoa, and general fungi were analyzed using a previously described quantitative PCR method [12–16]. Forty nanograms of extracted DNA was mixed with primers for the 16S rDNA region of target bacteria or the ITS region of eukaryotic microbes and amplified using SYBR Premix Ex Taq (TaKaRa Bio, China) and the LightCycler 480 Real-Time PCR System (Roche, Mannheim, Germany). The PCR mixtures were pre-incubated at 95°C for 5 min, denatured at 95°C for 10 s, annealed at 58°C for 10 s, and extended at 70°C for 10 s. After 45 cycles of amplification, a melting point test was performed. The annealing temperatures for individual primers varied depending on the primer sequences. The PCR amplicon was inserted into the TOP10 competent cell (Invitrogen, Carlsbad, CA, USA). After DNA extraction, serially diluted DNAs were amplified to create a standard curve for the absolute quantification of individual microorganisms.

### *In vitro* batch culture experiment

To investigate the effects of ruminal fluid on fermentation characteristics, two sets of *in vitro* experiments with the ruminal fluids obtained from steers were conducted. The substrates for the *in vitro* analysis were similar to the diets offered to the experimental steers, which received a mixture of 40% commercial concentrates and 60% rice straw. The concentrate and rice straw (Tables 1, 2) were oven-dried at 60°C for 3 days, milled to pass through a 1-mm sieve, and analyzed for chemical composition using the appropriate AOAC [17] and Van Soest methods [18].

### Incubation conditions

The medium [19] was dispensed into serum bottles under anaerobic conditions [20]. It was then infused with O_2_-free CO_2_ gas, and, simultaneously, strained rumen fluid was added as a microbial suspension (5%, v/v) using a syringe. The serum bottles were crimped with butyl rubber stoppers with aluminum seals and then incubated at 39°C for 0, 2, 4, 8, 12, and 24 h in a shaking water bath at 100 rpm. The experiments were performed in triplicate and conducted separately using the ruminal fluids obtained from each of three control and fasting animal. From each bottle, the gas volume was measured using a pressure detector (model PSGH-28PCCA, DECO Co., Seoul, Korea). Gas samples were collected in syringes with 3-way stopcocks from fermented gas-tight serum bottles, and CO_2_ and CH_4_ concentrations were estimated by gas chromatography (7890B GC, Agilent Technologies, Santa Clara, CA, USA). At the end of each incubation period, the supernatants were collected for pH determination and stored at −20°C for analyses of NH_3_-N [21], volatile fatty acids (VFAs) [22], and microbial protein synthesis [23]. Dry matter digestibility was also determined by filtering residues in a filter crucible, drying at 100°C, and weighing the resulting samples.

### Statistical analyses

The rumen microbial fermentation characteristics, including pH, gas production, NH_3_-N, microbial protein, VFA, acetate: propionate ratio, and CH_4_ at 0, 2, 4, 8, 12, and 24 h, and the quantity of the rumen microbial DNA extracted from the rumen contents were analyzed statistically using the LSMEANS statement of the MIXED procedure in the SAS program package [24]. Statistical differences were determined at p<0.05.

## RESULTS

**Figure 1:**
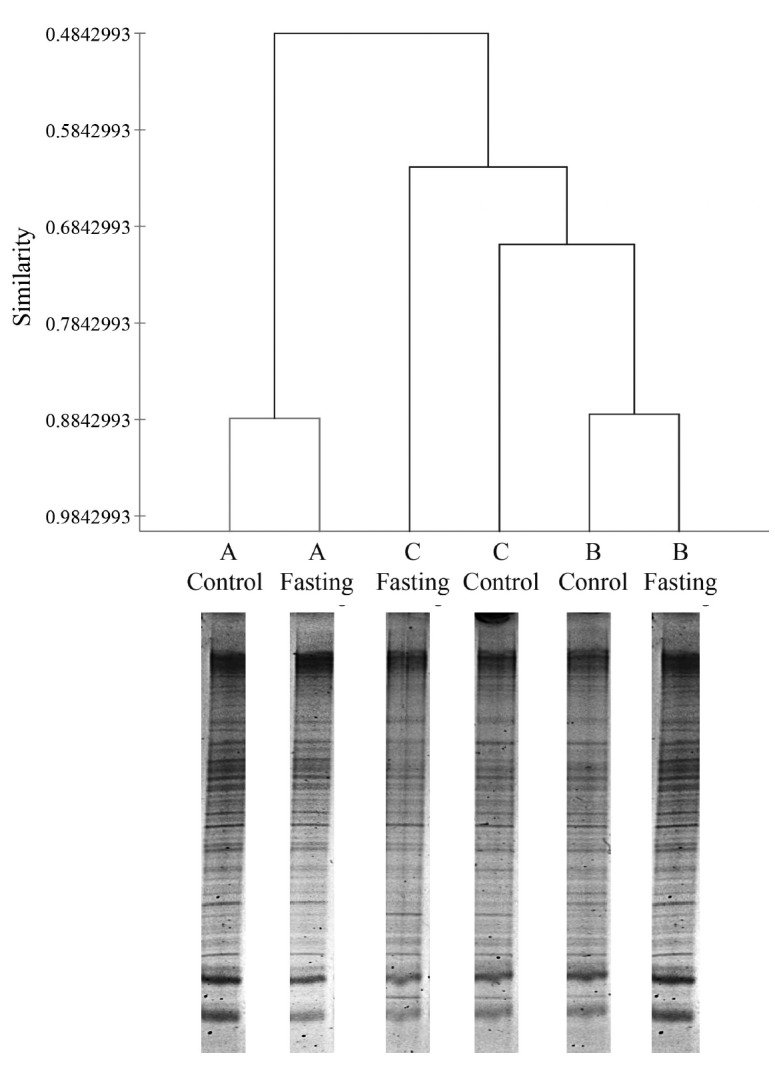
Effects of fasting on rumen microbiota based on the banding profile of denaturing gradient gel electrophoresis (A, B, and C indicate specific animals).

During fasting, steers did not show any abnormal symptoms until feed and water were reintroduced at the end of the study. The DGGE method was used to examine the differences in bacterial community between samples obtained before and after fasting (Figure 1). Although little variation was observed in fasting samples, we statistically compared the DGGE profiles of bacterial communities in control steers with those of each fasting steer. The variation and differences in community composition were analyzed by a clustering analysis of the DGGE gel profiles. Figure 1 shows that the DGGE profiles formed clusters representing each steer. The ruminal fluids of the control and fasted steers within treatments clustered together (steer A: 88.5% and steer B: 88.5%), except for steer C (67.6%), and a variation was observed among steers, which suggests that the rumen bacterial community changes slightly with short-term starvation (i.e., 24 h fasting). However, further analysis of the rumen microbiota showed marginal variation in the number of specific microorganisms (Table 3). The number of total bacteria (estimated as log copies) determined by qPCR was significantly (p<0.05) higher in the rumen contents of the control steers than in those of fasted steers. These differences reflect the differences between control and fasted steers in several specific rumen microorganisms, including *Anaerovibrio lipolytica* (p<0.05), *Eubacterium ruminantium* (p<0.05), *Prevotella albensis* (p<0.05), *Prevotella ruminicola* (p<0.05), and *Ruminobacter amylophilus* (p<0.05). However, the numbers of other major microorganisms in the rumen, such as protozoa, methanogenic archaea, and anaerobic fungi, did not differ between control and fasted animals (Table 3). Interestingly, substantial differences were found in the *in vitro* fermentation patterns when the rumen inoculum originated from control (no fasting) and fasted steers (Table 4). Although dry matter digestibility between control and fasted steers over the incubation period did not differ, total gas production, CH_4_, CO_2_, and VFA were higher (p<0.05) in the ruminal fluids of control animals than in the fluids from fasted animals throughout the incubation period.

## DISCUSSION

The rumen microbiota changes according to various factors, including diet, time after feeding, ruminant species, and physiological status of the animal, among others [25]. Our results suggest that when animals are fasted for a short period of time (i.e., 24 h), the rumen bacterial community changes slightly, but the number of total bacteria and specific populations differs between the rumen contents obtained before and after fasting. A previous study has shown that fasting impacts the gut microbiomes of tilapia, toads, geckos, quail, and mice [26]. According to the previous study, fasting induces changes in the microbiome in various host species and gut regions; however, microbial diversity increases with fasting in the colons of fish, toads, and mice. Presumably, this is explained by the limited nutrient supply in response to fasting, including water changes [27]. In addition, nutrient and water limitations may result in alterations in the relative abundance of ruminal microbes. This is interesting because large portions of ruminal microbes are solid-associated bacteria comprising 70% of the total bacteria in the rumen [7]. Therefore, when steers fast, even for just a short period (i.e., 24 h), limited feed particles in the rumen are likely to reduce the number of solid-associated bacteria. Indeed, the qPCR results in our study suggest that *Prevotella* and *Ruminobacter* decreased during fasting, whereas major cell wall-degrading bacteria (*R. albus*, *R. flavefaciens*, and *F. succinogenes*) did not change. Presumably, this may be attributed to slower degradation and longer retention of fiber in the rumen; cellulose-degrading bacteria could maintain their niches for longer than other bacteria. According to compartment theory, rumen microbes are classified by their distance from the feed particles or rumen epithelial tissue [7].

The planktonic bacterium *Prevotella* and weakly particle attached bacterium *Ruminobacter* are classified as compartment 1 and 2, respectively. The viability of those bacteria are more sensitive to the existence of available substrate than the other compartments which are tightly bind to substrate or rumen epithelial tissue. The decreased amounts of accessible substrates in the rumen caused decrease in the populations of planktonic and weakly particle attached bacteria during short-term fasting.

The objective of the present study was to examine the effect of fasting on rumen microbiota. Many laboratories worldwide use ruminal fluids as the microbial inoculum for *in vitro* fermentation studies, and differences in the rumen content properties may explain the differences in study outcomes. This concern is particularly important when comprehensive approaches, such as meta-analyses, are used for *in vitro* gas production and/or digestibility studies. Indeed, a recent meta-analysis of *in vitro* techniques indicated issues with these techniques for measuring gas and methane production and suggested “greater harmonization of analytical procedure” to improve our understanding of the results [28,29]. A previous study by Johnson [1] indicated the importance of the rumen inoculum from animals offered different forages when examining forage digestion *in vitro*, and more recently, excellent reviews have highlighted the critical requirements for *in vitro* studies [3]. According to Payne et al [4], gas production profiles are less variable at 4 or 8 h post-feeding than those either just before or 2 h post-feeding. This is in contrast with the findings of Menke and Steingass [6].

Our results suggest that the use of the ruminal fluid from fasting animals should be interpreted with caution. Although such findings do not preclude the use of the ruminal fluid from a slaughterhouse, additional care may be imperative, especially when comparisons are attempted among *in vitro* analyses. Extensive variation among animals was observed, and hence the use of single animals for *in vitro* or even *in situ* techniques may be suboptimal.

## Figures and Tables

**Table 1:** Ingredients of the concentrates (% of dry matter) and composition (% of dry matter) of concentrate and rice straw

Items	Concentrate	Rice straw
Ingredients		
Ground corn	3.02	
Wheat	2.10	
Soybean meal	2.40	
Rice bran	1.00	
Tapioca	17.8	
Sesame oil meal	1.40	
Palm kernel meal	41.08	
DDGS1)	22.0	
Molasses	5.00	
Condensed molasses soluble	1.00	
Salt	0.30	
Limestone	2.00	
CaCO_3_	0.7	
Minerals and vitamins mixture2)	0.7	
Chemical composition		
Dry matter	88.27	87.83
Crude protein	14.50	3.25
Ether extract	6.77	1.54
Crude fiber	12.68	28.40
Undegradable protein	7.36	-
Ash	7.49	15.68
Nitrogen-free extract	47.10	38.74
Non-fiber carbohydrate	19.83	1.66
Acid detergent fiber	23.08	44.13
Neutral detergent fiber	39.69	65.70
Total digestible nutrients	71.02	38.29

1) DDGS, dried distillers grain with solubles (USA).

2) Minerals and vitamins mixture, vitamin A 28,000 IU; vitamin D_3_ 4,000 IU; vitamin E 80 IU; Mn 80 ppm; Zn 100 ppm; Fe 70 ppm; Cu 50 ppm; Co 0.5 ppm; I 2.0 ppm; Se 1.0 ppm.

**Table 2:** Rumen fermentation parameters of experimental animals before and after fasting

Items	Control1)	Fasting1)	SEM	p-value
pH	5.88^B^	6.27^A^	0.0263	0.0005
NH_3_-N (mg/L)	14.50^B^	4.53^A^	0.5807	0.0003
Total volatile fatty acids (mmol/L)	77.50^B^	39.44^A^	2.8125	0.0007

SEM, standard error of the mean.

1) Control, inoculum (2 h after feeding); Fasting, inoculum (fasting for 24 h).

A,B Means in a row with different letters differ significantly (p<0.05).

**Table 3:** The effects of fasting on the rumen microbiota based on real-time polymerase chain reaction

Microorganisms	Control1)	Fasting1)	SEM	p-value

	---- Log copies/ng ----			
*Fibrobacter succinogenes*	8.03	8.05	0.045	ns
*Ruminococcus albus*	5.85	5.48	0.012	ns
*Ruminococcus flavefaciens*	3.83	3.72	0.078	ns
*Anaerovibrio lipolytica*	3.32^A^	3.02^B^	0.061	0.026
*Eubacterium ruminantium*	4.24^A^	4.05^B^	0.041	0.030
*Prevotella albensis*	6.08^A^	5.89^B^	0.031	0.012
*Prevotella ruminicola*	6.01^A^	5.80^B^	0.045	0.027
*Ruminobacter amylophilus*	5.83^A^	5.20^B^	0.117	0.020
*Streptococcus bovis*	3.66	3.36	0.134	ns
*Treponema bryantii*	6.61	3.57	0.047	ns
Total bacteria	11.52^A^	11.31^B^	0.006	0.002
Protozoa	4.73	4.75	0.104	ns
Methanogen	3.97	4.08	0.082	ns
Fungi	2.28	2.50	0.126	ns

SEM, standard error of the mean; ns, not significant.

1) Control, inoculum (2 h after feeding); Fasting, inoculum (fasting for 24 h).

A,B Means in a row with different letters differ significantly (p<0.05).

**Table 4:** Rumen fermentation characteristics determined using the rumen inoculum of non-fasted (control) and fasted (fasting) steers *in vitro*

Items	Incubation time (h)						

	0	2	4	6	8	12	24
pH value							
Control1)	7.18^B^	7.11^B^	7.03	7.06^A^	6.97	6.88	6.87
Fasting1)	7.31^A^	7.20^A^	7.03	6.98^B^	6.96	6.89	6.85
SEM	0.016	0.015	0.024	0.014	0.012	0.009	0.008
p-value	0.0001	0.0004	0.8975	0.0013	0.5215	0.1151	0.1607
Gas production (mL)							
Control1)	-	79.0^A^	125.3^B^	141.2^B^	161.0^A^	237.4^A^	269.6^A^
Fasting1)	-	14.4^B^	63.2^A^	108.8^A^	130.3^B^	202.5^B^	231.2^B^
SEM	-	9.82	12.06	4.90	4.86	5.09	4.50
p-value	-	0.0003	0.0022	0.0003	0.0004	0.0002	0.0001
Dry matter digestibility (%)							
Control1)	22.2	20.5	28.3	28.7	36.5	40.1	46.3
Fasting1)	22.2	20.3	28.7	30.3	33.0	41.9	44.2
SEM	1.19	2.23	1.75	0.82	1.91	1.16	1.25
p-value	0.9658	0.9312	0.0819	0.1837	0.2144	0.2714	0.2465
NH_3_-N concentration (mg/100 mL)							
Control1)	0.95^B^	1.41^B^	1.58	1.20	1.09	1.55	2.85^A^
Fasting1)	1.52^A^	2.27^A^	1.50	1.11	0.93	0.99	1.34^B^
SEM	0.07	0.27	0.26	0.12	0.13	0.28	0.46
p-value	0.0001	0.0419	0.8226	0.6081	0.4166	0.1804	0.0339
Microbial protein synthesis (mg/100 mL)							
Control1)	86.9^A^	85.0	78.7^B^	75.3	76.3	85.9^A^	100.6^A^
Fasting1)	62.0^B^	83.3	92.0^A^	81.0	77.8	77.7^B^	90.8^B^
SEM	1.38	1.94	1.63	2.17	1.70	1.66	2.47
p-value	0.0001	0.5632	0.0001	0.0797	0.5455	0.0032	0.0129
Total VFA concentration (mmol)							
Control1)	11.18^A^	13.87^A^	18.01^A^	23.52^A^	28.58^A^	40.69^A^	50.16^A^
Fasting1)	5.77^B^	7.12^B^	10.36^B^	13.69^B^	18.43^B^	35.52^B^	41.97^B^
SEM	1.651	0.695	0.746	0.926	1.081	1.164	1.049
p-value	0.0401	0.0001	0.0001	0.0001	0.0001	0.0063	0.001
Acetate/propionate ratio							
Control1)	3.39	2.48^B^	1.77^B^	1.53^B^	1.46^B^	1.46^B^	1.40^B^
Fasting1)	4.55	4.28^A^	3.80^A^	3.13^A^	2.57^A^	2.18^A^	2.24^A^
SEM	0.40	0.07	0.10	0.07	0.38	0.63	0.06
p-value	0.0579	0.001	0.0001	0.0001	0.0001	0.0001	0.0001
CH_4_ production (mL)							
Control1)	0.14	2.36^A^	8.17^A^	6.99^A^	9.74^A^	12.12^A^	15.52^A^
Fasting1)	0.10	0.44^B^	1.69^B^	3.24^B^	3.43^B^	4.07^B^	8.30^B^
SEM	0.29	0.30	1.07	0.41	0.48	1.15	0.63
p-value	0.3172	0.0004	0.0006	0.0001	0.0001	0.0001	0.0001
CO_2_ production (mL)							
Control1)	37.7^B^	75.5^B^	86.5	70.7	85.9^A^	75.3^A^	78.2^B^
Fasting1)	71.7^A^	86.8^A^	77.0	73.9	28.7^B^	42.8^B^	84.3^A^
SEM	6.79	1.91	7.51	7.72	5.03	9.52	1.75
p-value	0.0038	0.0010	0.3722	0.7659	0.0001	0.0336	0.0312

SEM, standard error of the mean; VFA, volatile fatty acids.

1) Control, inoculum (2 h after feeding); Fasting, inoculum (fasting for 24 h).

A,B Means in a column with different letters differ significantly (p<0.05).
